# Early Lineage Priming by Trisomy of *Erg* Leads to Myeloproliferation in a Down Syndrome Model

**DOI:** 10.1371/journal.pgen.1005211

**Published:** 2015-05-14

**Authors:** Ashley P. Ng, Yifang Hu, Donald Metcalf, Craig D. Hyland, Helen Ierino, Belinda Phipson, Di Wu, Tracey M. Baldwin, Maria Kauppi, Hiu Kiu, Ladina Di Rago, Douglas J. Hilton, Gordon K. Smyth, Warren S. Alexander

**Affiliations:** 1 The Walter and Eliza Hall Institute of Medical Research, Parkville, Victoria, Australia; 2 Department of Medical Biology, The University of Melbourne, Parkville, Victoria, Australia; 3 Department of Mathematics and Statistics, The University of Melbourne, Parkville, Victoria, Australia; 4 Centre for Cancer Research, Monash Institute of Medical Research, Monash University, Clayton, Victoria, Australia; 5 Department of Statistics, Harvard University, Cambridge, Massachusetts, United States of America; Cincinnati Children's Hospital Medical Center, UNITED STATES

## Abstract

Down syndrome (DS), with trisomy of chromosome 21 (HSA21), is the commonest human aneuploidy. Pre-leukemic myeloproliferative changes in DS foetal livers precede the acquisition of *GATA1* mutations, transient myeloproliferative disorder (DS-TMD) and acute megakaryocytic leukemia (DS-AMKL). Trisomy of the *Erg* gene is required for myeloproliferation in the Ts(17^16^)65Dn DS mouse model. We demonstrate here that genetic changes specifically attributable to trisomy of *Erg* lead to lineage priming of primitive and early multipotential progenitor cells in Ts(17^16^)65Dn mice, excess megakaryocyte-erythroid progenitors, and malignant myeloproliferation. Gene expression changes dependent on trisomy of *Erg* in Ts(17^16^)65Dn multilineage progenitor cells were correlated with those associated with trisomy of HSA21 in human DS hematopoietic stem and primitive progenitor cells. These data suggest a role for *ERG* as a regulator of hematopoietic lineage potential, and that trisomy of *ERG* in the context of DS foetal liver hemopoiesis drives the pre-leukemic changes that predispose to subsequent DS-TMD and DS-AMKL.

## Author Summary

An excess number of genes in trisomy on human chromosome 21 leads to the development of specific diseases in human Down syndrome. An excess copy of the gene, *ERG*, an ETS family transcription factor, has been implicated in abnormal blood system development in Down syndrome. In this study we show how trisomy of *Erg* in a murine Down syndrome model perturbs hematopoietic progenitor cells in a manner similar to that observed in human Down syndrome by inducing gene expression changes and lineage priming in early multi-potential progenitors. We show that the gene expression signature specifically attributable to trisomy of *Erg* in the murine model is strongly correlated with gene expression changes in human Down syndrome hematopoietic cells. The data suggest that *Erg* is an important regulator of megakaryocyte-erythroid lineage specification in multipotential hematopoietic cells and that trisomy of *Erg* in the context of DS prediposes to a transient myeloproliferative disorder and acute megakaryocyte leukaemia in a multi-step model of leukemogenesis.

## Introduction

Down syndrome (DS) is the commonest human aneuploidy [1]. DS infants with trisomy of human chromosome 21 (HSA21) are uniquely predisposed to a transient myeloproliferative disorder (DS-TMD) and acute megakaryocytic leukemia (DS-AMKL) [2]. DS-TMD, usually characterised by the presence of peripheral immature myeloblasts/megakaryoblasts and the variable involvement of other organs, is restricted to the neonatal period, spontaneously regresses and is the result of genetic co-operation between trisomy of HSA21 gene(s) with an acquired somatic mutation in *GATA1* in virtually all cases [3]. However, up to 30% of children will subsequently develop DS-AMKL, a malignancy clonally related to the preceding DS-TMD. Candidate gene analysis and genome-wide exome sequencing have identified somatic mutations and deletions implicated in the progression of DS-TMD to DS-AMKL, in genes including *JAK1*, *JAK2*, *JAK3*, *FLT3*, *TP53*, *TRIB1*, *MPL*, *EZH2*, *APC*, *PARK-2*, *PACRG*, *EXT1*, *DLEC1* and *SMC3*, and further suggested that *GATA-1* mutations alone in the context of HSA21 trisomy were sufficient for development of DS-TMD [4–12].

Preceding acquisition of *GATA1* mutations, human DS foetal livers exhibit perturbed hematopoiesis. Increased numbers and clonogenicity of hematopoietic stem (HSC) and progenitor cells, increased frequency of bi-potential megakaryocyte-erythroid progenitors, and reduced numbers of granulocyte-macrophage-committed progenitor cells have been described [13–15]. This perturbation must be attributed to a specific trisomic gene or genes on HSA21 that drive the pre-leukemic DS phenotype from which DS-AMKL and DS-TMD subsequently arise. Murine DS models with germline transmissible segmental trisomies of human or murine orthologues of HSA21 genes have allowed genetic analyses of the contributions of genes within the DS critical interval to specific DS phenotypes [16–19]. A well studied model is the Ts(17^16^)65Dn mouse, which is trisomic for orthologs of ~104 human chromosome 21 genes [17]. Ts(17^16^)65Dn mice display progressive myeloproliferation chracterised by thrombocytosis, megakaryocyte hyperplasia, dysplastic megakaryocytic morphology and myelofibrosis. Similarly, blasts with erythro-megakaryocytic features and myelofibrosis are commonly observed in organs affected by DS-TMD/AMKL, while DS foetal livers show increased numbers of bipotential megakaryocyte-erythroid progenitors with increased clonogenicity and megakaryocyte/erythroid potential as well as megakaryocytosis [13–15].

We previously implicated the ETS family transcription factor *ERG* as a critical HSA21 gene in DS hematopoietic disease by demonstrating that specific reversion of *Erg* gene dosage to functional disomy, while the other ~103 orthologs remained trisomic, abrogated the myelo-megakaryocytic proliferation in Ts(17^16^)65Dn mice [20]. *Erg* has previously been shown to be essential for normal hematopoietic stem cell function [21–23]. Moreover, *Erg* deregulation can cause erythro-megakaryocytic leukemia in mice [24,25], and is implicated in acute myeloid and lymphoid malignancy in humans. In t(16;21) AML that carry the *ERG/TLS-FUS* fusion, complex karyotype AML with amplification of 21q, normal karyotype adult AML, *MLL*-rearranged paediatric AML and in T-ALL, high levels of *ERG* expression correlate with poor prognosis [26–29].

The detailed mechanisms by which *Erg* contributes in trisomy to myeloproliferation in Ts(17^16^)65Dn mice, and whether molecular changes specifically driven by three copies of *Erg* in this model reflect those associated with human DS, remained to be elucidated. To address these questions, we detailed hematopoietic progenitor perturbations associated with malignant myeloproliferation in 4 month-old Ts(17^16^)65Dn mice, the youngest age at which myeloid progenitor abnormalities have been observed [30]. We then performed transcriptome analysis of hematopoieitic stem cell (HSC) and myeloid progenitor cell-enriched populations from the bone marrow of Ts(17^16^)65Dn mice to define the relevant biological and genetic changes by which trisomy predisposes to development of myeloproliferation in this DS model [20]. Transcriptome changes in multipotential progenitor cells that were attributed specifically to trisomy of *Erg* in the Ts(17^16^)65Dn mouse were then compared to expression changes due to trisomy of chromosome 21 (HSA21) in human DS CD34^+^CD38^-^ hematopoietic cells [31] to explore the role of *ERG* gene dosage in human disease.

## Results

### Trisomy of *Erg* in Ts(17^16^)65Dn mice drives changes to erythro-megakaryocytic progenitor cells similar to those in human DS

**Fig 1:**
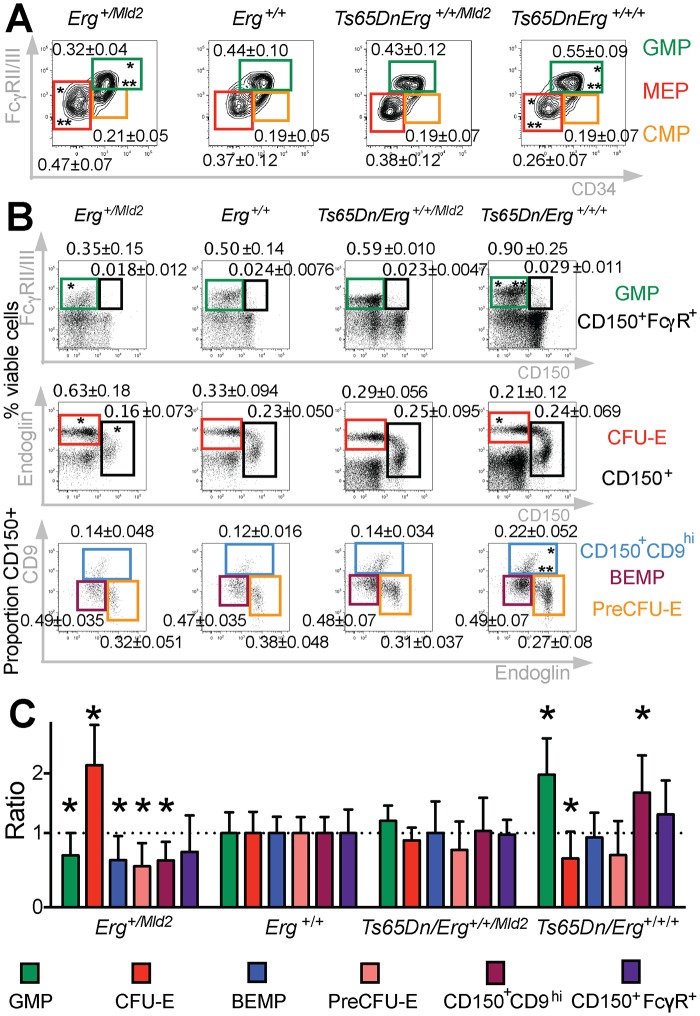
Perturbation of hematopoietic progenitors in the Ts(17^16^)65Dn DS mouse model due to trisomy of *Erg*. **A.** Relative proportions of GMP, CMP and MEP progenitor subsets within the Lineage^-^cKit^+^Sca1^-^ bone marrow fraction of *Erg* ^*+/Mld2*^ (n = 12; male = 9, female = 3), *Erg* ^*+/+*^ (n = 12; male = 4, female = 8), *Ts65Dn/Erg* ^*+/+/Mld2*^ (n = 10; male = 4, female = 6) and *Ts65Dn/Erg* ^*+/+/+*^ mice (n = 11; male = 7, female = 4). * *P* < 0.05 compared to *Erg* ^*+/+*^, ** *P* < 0.05 compared to *Ts65Dn/Erg* ^*+/+/Mld2*^ by Student's two-tailed t-test. **B & C.** Analysis of hematopoietic progenitor cells including bipotential erythroid-megakaryocyte progenitor populations as previously defined [33,34]. Representative flow cytometry profiles and gating strategies for analysis of cell populations from bone marrow of *Erg* ^*+/Mld2*^ (n = 13; male = 4, female = 9), *Erg* ^*+/+*^ (n = 10; male = 7, female = 3), *Ts65Dn/Erg* ^*+/+/Mld2*^ (n = 5; male = 4, female = 1) and *Ts65Dn/Erg* ^*+/+/+*^ (n = 7; male = 7) mice are shown. See S1 Table for immunophenotype definitions. (**B**) The mean number of each population is shown as the percentage of viable bone marrow cells or proportion of Lin^-^cKit^+^Sca1^-^CD150^+^ cells ± standard deviation. * *P* < 0.05 compared to *Erg* ^*+/+*^, ** *P* < 0.05 compared to *Ts65Dn/Erg* ^*+/+/Mld2*^. (**C**) The relative increase or decrease of each progenitor populations compared to wild-type (*Erg* ^*+/+*^) with mean and standard deviations shown, * *P* < 0.05 compared to *Erg* ^*+/+*^.

The stem-cell enriched lineage-negative cKit^+^Sca1^+^ (LSK) population has been previously shown to be expanded in trisomic Ts(17^16^)65Dn DS mice [20,30]. This population, which functionally resembles the expanded stem cell-enriched compartment in human DS foetal livers [15], was corrected to wild-type levels in Ts(17^16^)65Dn mice when *Erg* was specifically reduced from trisomy to functional disomy [20]. Moreover, it has been observed that, in addition to changes in stem cell numbers, bipotential erythroid-megakaryocyte progenitor populations are also perturbed in human DS [15]. We therefore sought to define the effects of trisomy on specific murine hematopoietic progenitor cells in the Ts(17^16^)65Dn DS model. Ts(17^16^)65Dn trisomic mice were crossed to mice carrying the non-functional Erg^*Mld2*^ allele as previously described [20]. The four resulting genotypes: mice trisomic for ~104 orthologs of human chromosome 21 genes including *Erg* (*Ts65Dn/Erg* ^*+/+/+*^), mice disomic for functional *Erg* and trisomic for the remaining ~103 genes in the trisomic segment (*Ts65Dn/Erg* ^*+/+/Mld2*^), euploid mice (*Erg* ^*+/+*^) and disomic mice with one functional *Erg* allele (*Erg* ^*+/Mld2*^), were analysed at 4 months of age for abnormalities in common myeloid progenitors (CMP), granulo-monocytic progenitors (GMP), and megakaryocyte-erythroid progenitors (MEP) as previously defined [32]. Consistent with previous data [30], increased numbers of GMPs and a deficit of MEPs were evident in *Ts65Dn/Erg* ^*+/+/+*^ mice (Fig 1A). These abnormalities were corrected in *Ts65Dn/Erg* ^*+/+/Mld2*^ mice (Fig 1A), suggesting that trisomy of *Erg* is specifically associated with perturbations of myeloid progenitors of several hematopoietic lineages in the Ts(17^16^)65Dn DS model.

Myeloid progenitors in *Ts65Dn/Erg* ^*+/+/+*^ mice were then examined in greater detail, with particular emphasis on the recently described series of progressively maturing BEMP, CD150^+^CD9^hi^ and CD150^+^FcγR^+^ bipotential erythroid-megakaryocyte progenitors [33,34], as well as pre-granulocyte macrophage (PreGM), granulocyte-macrophage (GMP), pre-colony forming unit erythroid (PreCFU-E) and colony forming unit erythroid (CFU-E) populations [33,35], defined in S1 Table (Fig 1B and 1C). Trisomic *Ts65Dn/Erg* ^*+/+/+*^ mice contained increased numbers of GMP and CD150^+^CD9^hi^ bipotential progenitor cells, and these were normalised in number when *Erg* was reduced to functional disomy in *Ts65Dn/Erg* ^*+/+/Mld2*^ mice. Conversely, the numbers of GMPs and bipotential megakaryocyte-erythroid progenitor populations (BEMP, CD150^+^CD9^hi^, CD150^+^FcγR^+^) were reduced in *Erg* ^*+/Mld2*^ mice haploinsufficient for functional *Erg*. Trisomic *Ts65Dn/Erg* ^*+/+/+*^ mice were noted to have significantly fewer CFU-E, an anomaly that was not evident in *Ts65Dn/Erg* ^*+/+/Mld2*^ mice and *Erg* ^*+/Mld2*^ mice had an expanded population of these late erythroid progenitors, supporting previous data that suggested *Erg* normally restrains terminal erythroid differentiation [22,24,25]. Consistent with the immunophenotypic analyses, in clonogenic assays, *Ts65Dn/Erg* ^*+/+/+*^ bone marrow demonstrated stimuli-specific increases in the numbers of granulocyte, macrophage and megakaryocyte colony-forming units (CFU). This myeloproliferation was moderated in *Ts65Dn/Erg* ^*+/+/Mld2*^ mice (Table 1). Together, these data support a role for *Erg* in regulation of multiple hematopoietic lineages. The bias toward megakaryocyte and away from erythroid progenitor formation that was specifically attributable to trisomy of *Erg* in Ts(17^16^)65Dn mice resembles progenitor abnormalities observed in pre-leukemic human DS foetal livers prior to acquisition of *GATA1* mutations, although human DS foetal livers exhibit reduced numbers of GMP, in contrast to the mouse model [15].

**Table 1:** Hematopoietic progenitor abnormalities in Ts65Dn(17^16^) bone marrow by semi-solid agar clonogenic colony assays.

G-CSF												
	G				GM			M			χ 2	
*Erg* ^*+/+*^ (n = 4)	13.5 ± 1.0				0.2 ± 0.5			0.0 ± 0.0			-	
*Erg* ^*+/Mld2*^ (n = 3)	6.3 ± 3.2 *				0.0 ± 0.0			0.0 ± 0.0			-	
*Ts65Dn/Erg* ^*+/+/+*^ (n = 6)	24.0 ± 4.9 * §				0.2 ± 0.4			0.2 ± 0.4			-	
*Ts65Dn/Erg* ^*+/+/Mld2*^ (n = 3)	9.0 ± 3.6 *				0.0 ± 0.0			0.0 ± 0.0			-	
**GM-CSF**												
	**G**		**GM**				**M**		**Eo**			χ 2
*Erg* ^*+/+*^ (n = 4)	24.2 ± 0.96		6.7 ± 2.6				44.0 ± 20.0		3.2 ± 2.1			-
*Erg* ^*+/Mld2*^ (n = 2)	13.5 ± 2.1		4.5 ± 0.7				56.5 ± 12.0		3.0 ± 0.0			2.8x10^-2^
*Ts65Dn/Erg* ^*+/+/+*^ (n = 6)	36.1 ± 12.6 §		12.3 ± 4.8				53.3 ± 24.8		1.2 ± 0.98			3.2x10^-3^
*Ts65Dn/Erg* ^*+/+/Mld2*^ (n = 3)	16.3 ± 2.1		3.3 ± 2.1				36.7 ± 15.9		2.0 ± 0.0			1.1x10^-1^
**IL-3**												
	**Blast**	**G**		**GM**			**M**	**Eo**		**Meg**		χ 2
*Erg* ^*+/+*^ (n = 4)	3.5 ± 2.4	31.5 ± 4.9		11.7 ± 3.9			15.2 ± 5.3	3.7 ± 0.96		5.5 ± 2.6		-
*Erg* ^*+/Mld2*^ (n = 3)	4.7 ± 3.8	21.3± 12.6		8.3 ± 3.0			17.7 ± 10.2	2.7 ± 2.9		4.7 ± 1.5		3.6x10^-1^
*Ts65Dn/Erg* ^*+/+/+*^ (n = 5)	5.8 ± 3.6	31.2 ± 9.4		12.4 ± 4.0			31.8 ± 17.9 * §	1.4 ± 0.9		9.8 ± 8.3		1.8x10^-4^
*Ts65Dn/Erg* ^*+/+/Mld2*^ (n = 3)	3.3 ± 3.5	23.6 ± 9.0		10.0 ± 6.2			15.6 ± 7.6	1.3 ± 1.5		3.3 ± 1.5		4.6x10^-1^
**SCF+IL3+EPO**												
	**Blast**	**G**		**GM**		**M**		**Eo**		**Meg**		χ 2
*Erg* ^*+/+*^ (n = 4)	7.5 ± 5.1	34.5 ± 15.8		16.2 ± 4.3		27.5 ± 9.8		2.0 ± 1.4		13.0 ± 3.1		-
*Erg* ^*+/Mld2*^ (n = 3)	5.3 ± 0.6	24.3 ± 7.3		17.3 ± 4.2		29.3 ± 8.0		1.7 ± 0.6		11.3 ± 4.0		5.4x10^-1^
*Ts65Dn/Erg* ^*+/+/+*^ (n = 6)	19.7± 18.3	41.3 ± 12.5§		19.5 ± 6.2		48.0 ± 15.4 * §		1.3 ± 1.5		29.5 ± 15.8 *		2.8x10^-11^
*Ts65Dn/Erg* ^*+/+/Mld2*^ (n = 3)	6.0 ± 5.3	27.0 ± 5.6		15.0 ± 4.0		23.3 ± 11.0		1.7 ± 0.6		17.7± 3.2		4.9x10^-1^

Numbers of colonies from 25,000 unfractionated bone marrow cells cultured in granulocyte colony-stimulating factor (G-CSF, 10^3^U/mL), granulocyte-macrophage colony-stimulating factor (GM-CSF, 10^3^U/mL), interleukin-3 (IL3, 10ng/mL), stem-cell factor (SCF, 100 ng/mL), erythropoietin (EPO, 2U/mL), as indicated, with the type and number of colonies scored after 7 days. The number of biological replicates per genotype is given. Mean and standard deviation shown.

* = *P* < 0.05 for comparison with *Erg* ^*+/+*^;

^§^ = *P* < 0.05 for comparison with *Ts65Dn/Erg* ^*+/+/Mld2*^ mice by unprotected Fisher’s Least Significant Difference.

χ2 for difference for a given genotype compared to diploid control *Erg* ^*+/+*^ comparing all colony numbers using rounded means shown. Blast, blast colony; G, granulocyte; GM, granulocyte-macrophage; M, macrophage; Eo, eosinophil; Meg, megakaryocyte colony.

### Trisomy of *Erg* drives gene expression changes in multipotential progenitor cell populations

We undertook gene expression profiling of prospectively isolated LSK, CMP, GMP and MEP populations from *Ts65Dn/Erg* ^*+/+/+*^, *Ts65Dn/Erg* ^*+/+/Mld2*^, *Erg* ^*+/+*^ and *Erg* ^*+/Mld2*^ mice. Gene expression changes within each cell population that were attributable to changes in functional *Erg* gene dosage were determined by specific pair-wise comparisons using linear modeling and empirical Bayes moderated t-statistics [36]. Changes due to full trisomy of all ~104 syntenic genes were inferred from comparison of *Ts65Dn/Erg* ^*+/+/+*^ versus *Erg* ^*+/+*^ mice, trisomy-induced changes specifically attributable to trisomy of *Erg* were evident from comparison of *Ts65Dn/Erg* ^*+/+/+*^ versus *Ts65Dn/Erg* ^*+/+/Mld2*^ (effects specific to *Erg* trisomy), changes due to trisomy with two copies of functional *Erg* (effects due to non-*Erg* gene trisomy) emerged from comparison of *Ts65Dn/Erg* ^*+/+/Mld2*^ versus *Erg* ^*+/+*^, and changes due to Erg haplo-insufficiency from comparison of *Erg* ^*+/Mld2*^ versus *Erg* ^*+/+*^.

**Fig 2:**
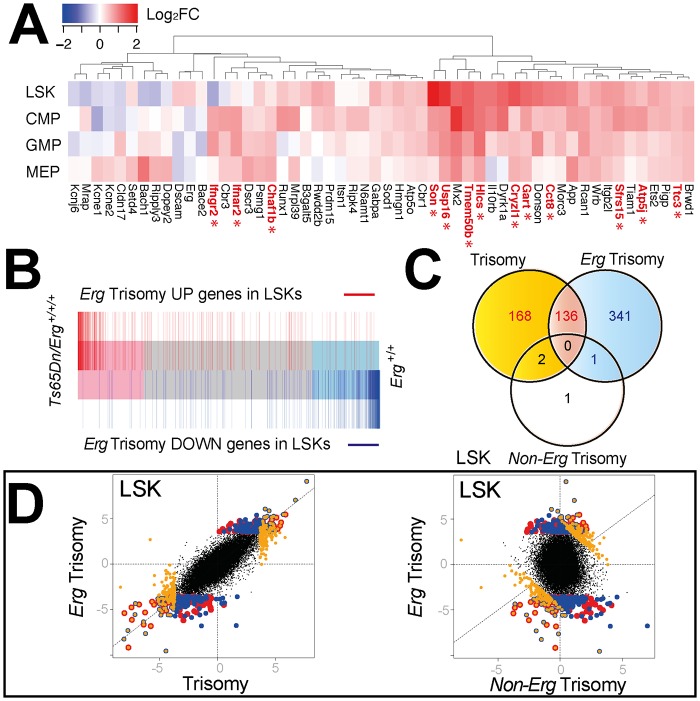
Comparison of gene expression changes due to full trisomy and specifically to trisomy of *Erg* in Ts(17^16^)65Dn LSKs. **A.** Differential expression of murine orthologs of HSA21 trisomic genes in Ts65 (17^16^)Dn hematopoietic cells [51]. Heatmap diagram of log_2_ fold expression changes for genes with detectable expression in microarray data from the LSK, CMP, GMP and MEP cells for comparison between *Ts65DnErg* ^*+/+/+*^ and *Erg* ^*+/+*^ genotypes. Trisomic genes with significantly upregulated gene expression in Ts65(17^16^)Dn cells using a false discovery rate of 5% are indicated in red with an asterix. **B.** Barcode plot demonstrating distribution of differentially expressed genes due specifically to trisomy of *Erg* (pairwise comparison *Ts65Dn/Erg* ^*+/+/+*^ versus *Ts65Dn/Erg* ^*+/+/Mld2*^; red bars: upregulated genes; blue bars: downregulated genes) relative to gene expression changes due to overall trisomy (pairwise comparison *Ts65Dn/Erg* ^*+/+/+*^ versus *Erg* ^*+/+*^) in LSK cells. **C.** Venn diagram of differentially regulated genes in LSK cells due to the effect of overall trisomy (*Ts65Dn/Erg* ^*+/+/+*^ versus *Erg* ^*+/+*^), trisomy of *Erg* (*Ts65Dn/Erg* ^*+/+/+*^ versus *Ts65Dn/Erg* ^*+/+/Mld2*^) and *non-Erg* trisomy (Ts65Dn/Erg^*+/+/Mld2*^ versus *Erg* ^*+/+*^). Intersect of differentially expressed genes by t-stastistic using an FDR of < 5%; (red) intersect of differentially expressed genes due to trisomy and *Erg* trisomy; (yellow) non-intersecting differentially expressed genes due to trisomy; (blue) non intersecting differentially expressed genes due to trisomy of *Erg*. **D.** Dot plot of differentially expressed genes in LSK cells by t-statistic comparing expression differences resulting from overall trisomy (*Ts65Dn/Erg* ^*+/+/+*^ versus *Erg* ^*+/+*^) to those specifically attributable to *Erg* trisomy (*Ts65Dn/Erg* ^*+/+/+*^ versus *Ts65Dn/Erg* ^*+/+/Mld2*^, left panel) and *Erg* trisomy compared to expression changes specific for non*-Erg* genes in trisomy (Ts65Dn/Erg^*+/+/Mld2*^ versus *Erg* ^*+/+*^, right panel). Genes identified in the Venn diagram (Fig 2C) are shown using the same color scheme suggesting a high degree of correlation between differentially expressed genes due to trisomy and *Erg* trisomy in LSK cells.

We initially explored the expression in LSK, CMP, GMP and MEP cells of the ~104 HSA21 orthologs present in trisomy in *Ts65Dn/Erg* ^*+/+/+*^ mice compared with their expression in *Erg* ^*+/+*^ cells. Increased expression of these genes in trisomc cells varied and was also dependent on the specific hematopoietic cell type examined (Fig 2A). At a false discovery rate of 5% across the entire transcriptome, *Son*, *Usp16*, *Cyrzl1*, *Gart*, *Cct8* were upregulated in LSK cells, *Sfrs15*, *Ifngr2*, *Ifnar2*, *Gart*, *Atp5j* in CMPs, and *Gart*, *Chaf1b*, *Hlcs*, *Ttc3* in GMPs of Ts(17^16^)65Dn mice (Fig 2A and S2 Table).

The greatest number of gene expression changes due to full trisomy (*Ts65Dn/Erg* ^*+/+/+*^ versus *Erg* ^*+/+*^) or specifically to Erg trisomy (*Ts65Dn/Erg* ^*+/+/+*^ versus *Ts65Dn/Erg* ^*+/+/Mld2*^) occurred in the LSK compartment, while gene expression changes attributable to trisomy of non*-Erg* genes in the interval (*Ts65Dn/Erg* ^*+/+/Mld2*^ versus *Erg* ^*+/+*^) were fewer and occurred principally in MEP and GMP populations (Table 2). In addition, in LSK cells there was a strong degree of overlap in differentially expressed genes due to full trisomy and specifically due to trisomy of *Erg*, both in up- and down-regulated genes (Fig 2B, 2C and 2D).

**Table 2:** Differential gene expression due to full trisomy, attributable specifically to *Erg* trisomy, and trisomy of non*-Erg* genes.

		LSK	CMP	GMP	MEP
**Full trisomy** Ts65Dn/Erg^+/+/+^ versus Erg^+/+^	*UP*	147	35	29	90
	*NS*	22068	22318	22310	22212
	*DOWN*	159	21	35	72
***Erg* trisomy specifically** Ts65Dn/Erg^+/+/+^ versus Ts65Dn/Erg^+/+/Mld2^	*UP*	217	2	2	4
	*NS*	21896	22369	22369	22364
	*DOWN*	261	3	3	6
**Non*-Erg* gene trisomy** Ts65Dn/Erg^Mld2/+/+^ versus Erg^+/+^	*UP*	2	1	19	27
	*NS*	22370	22370	22335	22323
	*DOWN*	2	3	20	24

Differential gene expression with the number of up-regulated and down-regulated probes (False discovery rate (FDR) of 0.05, NS = not significant), as a consequence of full trisomy (pairwise comparison *Ts65Dn/Erg* ^*+/+/+*^ versus *Erg* ^*+/+*^), trisomy of *Erg* (*Ts65Dn/Erg* ^*+/+/+*^ versus *Ts65Dn/Erg* ^*+/+/Mld2*^) and *non-Erg* gene trisomy (*Ts65Dn/Erg* ^*+/+/Mld2*^ versus *Erg* ^*+/+*^) in LSK, CMP, GMP and MEP hematopoietic cells (see [20], Fig 1A and S2 Table). Three biological replicates per cell type per genotype were obtained using pooled RNA derived from 2–4 mice (including male and female) per genotype per replicate, for a total of 48 arrays.

**Fig 3:**
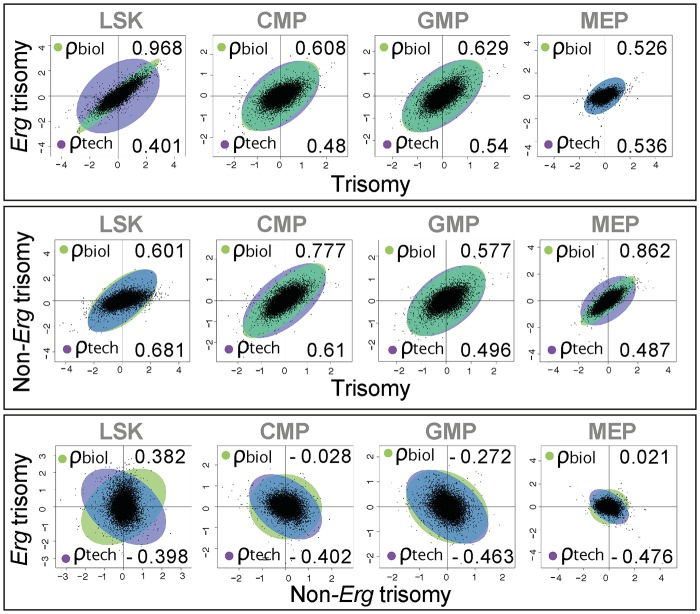
Biological correlation of gene expression changes using gene association analysis. Scatter plots by log_2_ fold gene expression changes in LSK, CMP, GMP and MEP cells comparing: top panel, changes due to full trisomy (*Ts65Dn/Erg* ^*+/+/+*^ versus *Erg* ^*+/+*^) with those specific for trisomy of *Erg* (*Ts65Dn/Erg* ^*+/+/+*^ vs *Ts65Dn/Erg* ^*+/+/Mld2*^); middle panel, expression changes due to full trisomy with those attributable to non*-Erg* genes in trisomy (*Ts65Dn/Erg* ^*+/+/Mld2*^ versus E*rg* ^*+/+*^); and lower panel changes attributable to *Erg* trisomy with those non*-Erg* genes in trisomy. Green ellipses indicate the strength of biological correlation (ρ_biol_) and blue ellipses show technical correlation (ρ_tech_) as estimated by GENAS.

Given the correlation of gene expression changes induced by full trisomy with those associated specifically with trisomy of *Erg*, a Genuine Association Analysis (GENAS) was undertaken. GENAS is a relatively new statistical technique that estimates the biological correlation between two differential expression profiles, correcting for any technical correlation and for the statistical uncertainty with which the fold changes are estimated [37,38]. Unlike the initial analysis in Fig 2B, GENAS does not depend on a significance cut-off and instead calculates an overall correlation using all expressed genes. In confirmation of the initial analyses, there was a very high degree of biological correlation in LSK cells between gene expression changes caused by trisomy of the full HSA21 syntenic segment and those associated specifically with trisomy of *Erg*. The strongest correlation between expression changes resulting from full trisomy and those due to trisomy of non-*Erg* genes occurred within MEP cells (Fig 3). Notably, the degree of biological correlation between gene expression changes attributable specifically to *Erg* trisomy and those due to trisomy of *non-Erg* genes was weak in all cell types analysed, suggesting gene expression changes induced specifically by trisomy of *Erg* were distinct from the combined effect of other genes within the Ts(17^16^)65Dn trisomic segment.

Together, these findings establish that in hematopoietic progenitor cells, *Erg* is the dominant influence within the Ts(17^16^)65Dn trisomic interval, three copies of which specifically drives gene expression changes that are evident primarily within multipotential hematopoietic progenitor populations rather than more lineage-restricted progenitors.

### Trisomy of *Erg* induces lineage priming in multipotential progenitor cells

We next sought to understand how gene expression changes induced specifically by trisomy of *Erg* could lead to perturbations of hematopoietic progenitors and the myeloproliferative phenotype observed in Ts(17^16^)65Dn mice. To do this, we undertook an expression signature analysis of the expression changes in the *Ts65Dn/Erg* ^*+/+/+*^ versus *Ts65Dn/Erg* ^*+/+/Mld2*^ cells using curated gene sets from the Molecular Signatures database (MSigDB version 2.5) and pathway gene sets defined by the BioCarta, KEGG and Reactome databases. A ROAST test was conducted for each signature gene set. ROAST is a gene set test suitable for small samples and linear models that accounts for inter-gene correlation [38,39]. ROAST evaluates whether the overall expression signature defined by a gene set is up- or down-regulated within a specific comparison. This analysis found that expression signatures associated with progenitor cells of specific hematopoietic lineages, including granulocyte-monocyte progenitors, megakaryocytes, platelets and platelet processes, were significantly upregulated in the *Ts65Dn/Erg* ^*+/+/+*^ cells (ROAST *P*-value < 0.05, S3 Table). By contrast, signatures associated with myeloproliferation were not significantly enriched in *Ts65Dn/Erg* ^*+/+/+*^ versus *Ts65Dn/Erg* ^*+/+/Mld2*^ LSK cells.

Given these findings, and as we had observed perturbations in specific hematopoietic progenitor populations in *Ts65Dn/Erg* ^*+/+/+*^ bone marrow (Fig 1 and Table 1), we examined whether the gene expression changes specific to trisomy of *Erg* in Ts(17^16^)65Dn mice could be interpreted in terms of cell lineage priming. We generated gene expression signatures that define specific cell populations: LSK, GMP and their precursors (Pre-GM Flt3^+^, Pre-GM Flt3^-^), megakaryocytes and their progenitors (BEMP, CD150^+^ CD9^hi^, CD150^+^ FcγR^+^), and CFU-E and pre-CFU-E. An expression signature was defined for each cell population by compiling the significantly up-regulated genes in that cell type compared to the average of all the other populations, together with the magnitude of the up-regulation for each gene as measured by the moderated t-statistic (S4 Table). ROAST tests were then conducted, with genes weighted by their magnitude of change, to determine whether these signatures were associated with the gene expression changes due to full trisomy (*Ts65Dn/Erg* ^*+/+/+*^ versus *Erg* ^*+/+*^), the specific effects of *Erg* trisomy (*Ts65Dn/Erg* ^*+/+/+*^ versus *Ts65Dn/Erg* ^*+/+/Mld2*^), the effects of trisomy of non-*Erg* genes (*Ts65Dn/Erg* ^*+/+/Mld2*^ versus *Erg* ^*+/+*^) and *Erg* haploinsufficiency (*Ts65Dn/Erg* ^*+/+*^ versus *Erg* ^*+/Mld2*^) in LSK, CMP, GMP and MEP populations.

**Fig 4:**
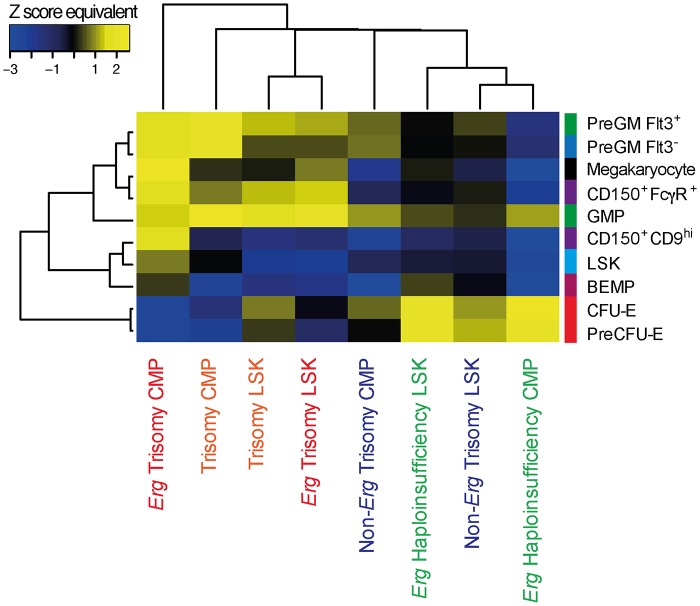
Hematopoietic lineage specific gene signatures induced by *Erg* trisomy in Ts(17^16^)65Dn LSK and CMP cells. Enrichment of LSK, myeloid progenitor and megakaryocyte gene signatures in the gene expression changes due to complete trisomy (defined by the comparison of gene expression in *Ts65Dn/Erg* ^*+/+/+*^ versus *Erg* ^*+/+*^ cells), the gene expression changes attributable specifically to *Erg* trisomy (*Ts65Dn/Erg* ^*+/+/+*^ versus *Ts65Dn/Erg* ^*+/+/Mld2*^), those due to trisomy of non*-Erg* genes (*Ts65Dn/Erg* ^*+/+/Mld2*^ versus E*rg* ^*+/+*^); and those due to *Erg* haploinsufficiency (*Ts65Dn/Erg* ^*+/+*^ vs *Ts65Dn/Erg* ^*+/Mld2*^) in LSK and CMP populations. Heatmap by Pearson correlation of Z score equivalents derived from ROAST *P*-values using progenitor gene signatures weighted by moderated t-statistic is shown (see S4 Table, Materials and Methods). Yellow indicates upregulation of cell type-specific gene signatures (positive Z score equivalent) and blue downregulation of these signatures (negative Z score equivalent). A higher absolute Z score equivalent value represents more significant enrichment.

Signatures of more committed progenitors, particularly multipotential PreGM Flt3^-^ cells, granulo-monocytic progenitors (PreGM Flt3^+^ and GMP), bipotential megakaryocyte-erythroid progenitors (CD150^+^CD9^hi^ and CD150^+^FcγR^+^) and megakaryocytes, were enriched in the gene expression changes due to trisomy of *Erg* in LSK cells, and this was also evident in CMPs by this comparison (Fig 4). In LSK cells, this was accompanied by downregulation of the normal LSK gene signature indicative of a more differentiated profile (Fig 4). In contrast, erythroid progenitor signatures (PreCFU-E and CFU-E) were enriched in the gene expression changes caused by haploinsufficiency of functional *Erg* in LSK and CMP cells (Fig 4). Strikingly, these genetic changes attributable specifically to trisomy of *Erg* in Ts(17^16^)65Dn LSKs and CMPs were concordant with the increased numbers of GMP and megakaryocyte-committed cells and fewer erythroid progenitors that were evident in *Ts65Dn/Erg* ^*+/+/+*^ but not *Ts65Dn/Erg* ^*+/Mld2+*^ mice, as well as the relative enrichment of erythroid progenitors in mice with *Erg* haploinsufficiency (Fig 1).

Finally, we investigated the expression of genes implicated by somatic mutations and deletions in the progression of DS-TMD to DS-AMKL. Of these genes, expression of the murine orthologues for *JAK1*, *JAK2*, *JAK3*, *FLT3*, *TP53*, *TRIB1*, *MPL*, *EZH2*, *APC*, *EXT1* and *SMC3* was detected in the microarray data from LSK, CMP, GMP or MEP cells. While modest differences were evident in expression of some of these genes in comparisons between cells from *Ts65Dn/Erg* ^*+/+/+*^ and *Erg* ^*+/+*^ mice, differential expression of these genes did not reach statistical significance (S1 Fig and S2 Table).

Thus, the data support a model in which gene expression changes attributable specifically to trisomy of *Erg* in Ts(17^16^)65Dn mice occur primarily in multipotential hematopoietic cells with priming for specific hematopoietic progenitor lineages leading to myeloid progenitor perturbation, myeloproliferative changes and megakaryocytosis.

### *Erg-*trisomy gene signatures in Ts(17^16^)65Dn LSKs correlate with gene expression changes in human DS CD34^+^CD38^-^ bone marrow cells

**Fig 5:**
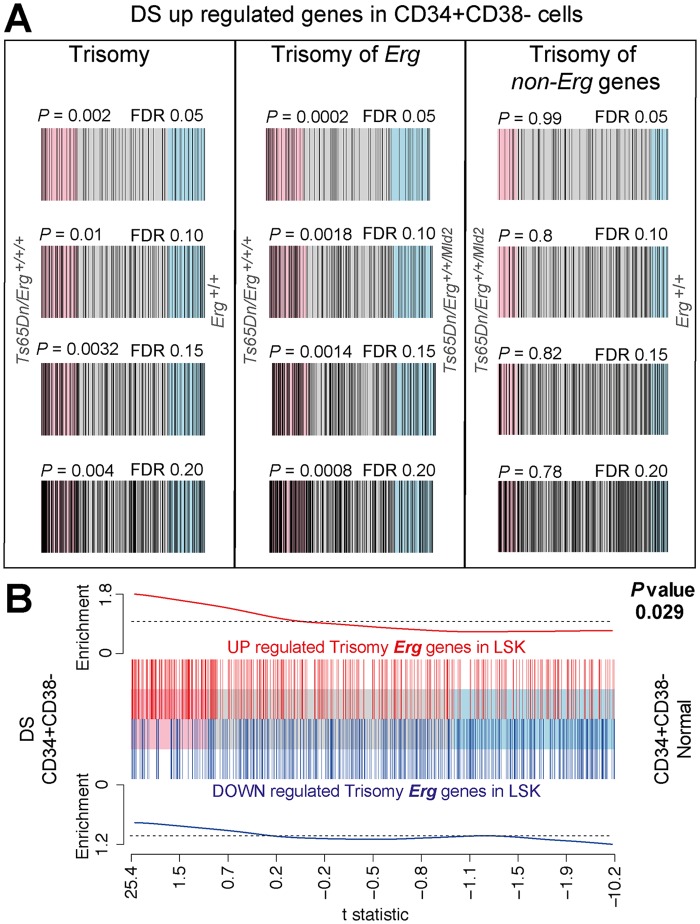
Human DS Lin^-^CD34^+^CD38^-^ HSPC gene expression changes correlate with changes due to trisomy of *Erg* in Ts(17^16^)65Dn LSK cells. **A.** Barcode plots of upregulated genes in human DS Lin^-^CD34^+^CD38^-^ stem and progenitor cells (HSPC) relative to diploid HSPCs using a FDR of 0.05, 0.10, 0.15 and 0.20 (see S5 Table) compared to gene expression changes in Ts(17^16^)65Dn LSK cells due to full trisomy defined by the comparison of gene expression in *Ts65Dn/Erg* ^*+/+/+*^ versus *Erg* ^*+/+*^ cells, (left panels), changes attributable specifically to *Erg* trisomy (*Ts65Dn/Erg* ^*+/+/+*^ versus *Ts65Dn/Erg* ^*+/+/Mld2*^, middle panels), and those due to trisomy of non*-Erg* genes (*Ts65Dn/Erg* ^*+/+/Mld2*^ versus E*rg* ^*+/+*^, right panels). Human DS HSPC genes (black bars) are upregulated in Ts(17^16^)65Dn LSK gene expression changes attributable specifically to trisomy of *Erg* (middle panels) but not due to trisomy of non*-Erg* genes (right panels). Rotation gene set test ROAST *P-*values shown. **B.** Barcode plot showing correlation between gene expression changes in Ts(17^16^)65Dn LSK cells due to *Erg* trisomy (from comparison of *Ts65Dn/Erg* ^*+/+/+*^ versus *Ts65Dn/Erg* ^*+/+/Mld2*^) with gene expression changes in human DS HSPCs relative to diploid cells. Horizontal axis shows moderated t-statistic values for the human DS HSPC versus diploid HSPCs comparison. Red bars show upregulated genes and blue bars downregulated genes resulting specifically from *Erg* trisomy in Ts(17^16^)65Dn LSK cells (FDR < 0.05, S2 Table). Red and blue worms show relative enrichment of up and downregulated genes respectively (ROAST *P-*value = 0.029 for upregulated genes).

Transcriptome profiling of human DS Lin^-^CD45^+^CD34^+^CD38^-^ bone marrow cells enriched for HSCs and multipoential hematopoietic progenitors that had been previously combined with transcriptomes of DS neurospheres derived from foetal cortical precursors, demonstrated differences in gene expression when compared to diploid controls [31]. We sought to establish if DS Lin^-^CD45^+^CD34^+^CD38^-^ bone marrow cells specifically demonstrated gene expression changes associated with trisomy of HSA21 when compared to diploid controls, and if these were related to differential gene expression due to *Erg* trisomy in LSKs in the Ts(17^16^)65Dn DS model. From the published data [31], we derived gene expression signatures for human DS HSC and multipotential progenitor cells that were induced by trisomy of HSA21 (S5 Table). We then used ROAST to compare these human DS gene signatures to gene expression changes induced by full trisomy in Ts(17^16^)65Dn LSK cells, as well as those specific to trisomy of *Erg* and those due to trisomy of non*-Erg* genes (Fig 5). The human DS gene signatures were strongly and significantly correlated with gene expression changes due specifically to *Erg* trisomy in Ts(17^16^)65Dn LSK cells, but not with those attributable to trisomy of non*-Erg* genes (Fig 5A). The complementary analysis was then undertaken. ROAST tests showed that gene expression changes specifically induced by trisomy of *Erg* in Ts(17^16^)65Dn LSK cells significantly correlated with gene expression changes due to trisomy of HSA21 in human DS Lin^-^CD34^+^CD38^-^ cells (Fig 5B). This genetic data supports the role of *ERG* as a key gene on HSA21 which in trisomy, drives the hematopoietic phenotype in human DS.

## Discussion

We show here, in comparisons between *Ts65Dn/Erg* ^*+/+/+*^ mice, in which a ~104-gene interval syntenic to HSA21 is present in trisomy, and their *Ts65Dn/Erg* ^*+/+/Mld2*^ counterparts, in which the *Erg* gene within this interval is specifically reduced to functional disomy, that trisomy of *Erg* is specifically required for the characteristic perturbations in specific hematopoietic progenitor cell populations in the Ts(17^16^)65Dn DS model. Unlike transgenic and retroviral models of induced *Erg* expression, the use of the non-functional *Erg* ^*Mld2*^ allele in this model allows accurate characterisation of effects of functional *Erg* gene dosage while expression is endogenously regulated. The hematopoietic changes in Ts(17^16^)65Dn mice reflected the pre-leukemic changes observed in human DS foetal livers prior to acquisition of *GATA-1* or other somatic mutations. While these analyses do not exclude contribution from other genes within the trisomic interval, they clearly establish that three copies of *Erg* is specifically required to drive transcriptome changes in HSCs and early multipotential progenitors in Ts(17^16^)65Dn mice. Moreover, the data provide a mechanism by which deregulation of *Erg* leads to perturbation of myeloid progenitor populations, myeloproliferation and megakaryocytosis in trisomy. Our findings show that perturbation of functional *Erg* gene dose by trisomy or by haploinsufficiency, is associated with lineage specific gene expression changes and "lineage priming" of multipotential hematopoietic progenitor cells. Notably, the effects specific to trisomy of *Erg* on gene expression and progenitor cell alterations in Ts(17^16^)65Dn mice were strongly reflected in *Erg* ^*+/Mld2*^ mice, but in the opposite direction. Finally, we demonstrate that the gene signature specifically associated with trisomy of *Erg* in Ts(17^16^)65Dn LSK cells is directly related to gene expression changes in human DS Lin^-^CD34^+^CD38^-^ cells enriched for HSCs and early multipotential progenitors. These data support a key role for *ERG* as a critical gene in trisomy of HSA21 that drives hematopoietic changes in human DS.

**Fig 6:**
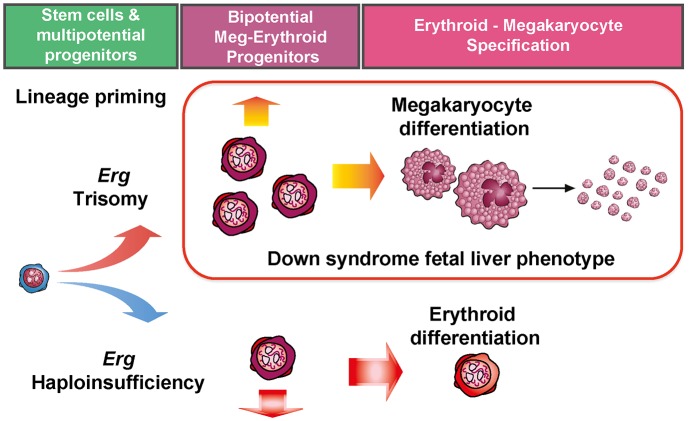
Perturbation of functional *Erg* gene dosage induces lineage specific gene expression changes in multipotential progenitor cells. Trisomy of *Erg* in Ts(17^16^)65Dn mice and *Erg* haploinsufficiency in *Mld2* mutants induce lineage specific gene expression changes in multipotential hematopoietic progenitor cells. Trisomy of *Erg* leads to an increase in bipotential erythroid-megakaryocytic progenitors, erythroid maturation block, megakaryocytosis, and the pre-leukemic human DS foetal liver phenotype. Haploinsufficiency of *Erg* results in in fewer bipotential progenitors and an increase in committed erythroid progenitors as observed in the *Erg* ^*+/mld2*^ mouse.

Previous analyses established that *Erg* is an important regulator of HSC self-renewal after establishment of definitive hematopoiesis in the embryo [23] and in emergency hematopoiesis in adult HSCs [21,22]. The data presented here imply additional roles for *Erg* for lineage priming in early multipotential hematopoietic cells which affects subsequent myeloid lineage development (Fig 6). This includes granulocyte-monocyte progenitors and bipotential erythroid-megakaryocyte progenitors, with effects on megakaryocyte and erythroid lineage specification being of particular relevance. In these latter roles, haploinsufficiency of functional *Erg* resulted in fewer bipotential erythroid-megakaryocyte progenitors and a propensity toward CFU-E formation, consistent with previous transplantation studies which demonstrated a bias toward erythroid lineage formation from *Erg* ^*+/Mld2*^ HSCs [22]. Trisomy specifically related to *Erg* in the Ts(17^16^)65Dn model led to expansion of bipotential megakaryocyte-erythroid progenitors, fewer committed erythroid progenitors and megakaryocytosis [20]. This finding is consistent with changes in human DS foetal livers [15] and other independent murine models of *Erg* overexpression [24,40]. Indeed, aberrant megakaryocyte-erythroid differentiation and erythroid maturation block may be a potential unifying mechanism for *Erg* in predisposing to acute bipotential megakaryocytic-erythroid leukemia [25]. The selective expansion of CD150^+^CD9^hi^ cells within bipotential megakaryocyte-erythroid progenitor compartment in Ts(17^16^)65Dn mice is similar to that observed in murine models of thrombopoietin driven myeloproliferation [33,34], providing additional evidence that this progenitor population correlates with the degree of megakaryocytosis in disease models. It was also noted that Ts(17^16^)65Dn mice exhibited increased numbers of GMP, while human DS foetal livers exhibit reduced numbers of these progenitors [15]. This discrepancy may by attributable to the comparatively low level of endogenous *Erg* expression in murine models relative to human hematopoietic cells [40]. Indeed, in a transgenic model of *ERG* overexpression, reduction in GMP number was observed in murine foetal livers [40], suggesting the level of gene expression and the hematopoietic stage is important in explaining this apparently discordant phenotype.

It is notable that the expansion of the LSK compartment in *Ts65Dn/Erg* ^*+/+/+*^ mice, which is specifically dependent on trisomy of *Erg* [20], reflects expansion of HSPCs in DS foetal livers [15], and was associated with downregulation of the LSK gene signature and up-regulation of progenitor cell-specific gene expression patterns, making trisomic LSKs less "stem cell-like". This suggests that an excess of *Erg* favors progenitor specification at the expense of HSC maintenance. This finding is in keeping with the increased multi-potential pre-progenitor cell frequency in *Ts65Dn/Erg* ^*+/+/+*^ bone marrow [20] and the increased propensity to form "blast-my" colonies upstream from CFU-GEMM in DS foetal liver [15]. These observations also argue against increased *Erg* gene dose inducing a program of self-renewal as a leukemogenic mechanism in the development of DS-AMKL and DS-TMD. Indeed, competitive transplantation assays using the Ts1Rhr trisomic murine model of DS, which carries a trisomic segment that includes the *Erg* gene, demonstrated fewer competitive repopulating units compared to disomic controls [41]. Nevertheless, the observation that the multipotential PreGMFlt3^-^ progenitor signature was also upregulated in *Ts65Dn/Erg* ^*+/+/+*^ CMPs relative to *Ts65Dn/Erg* ^*+/+/Mld2*^ suggests that in trisomy, *Erg* allows maintenance of multipotential progenitor potential while promoting specific lineage commitment toward megakaryocyte and granulocyte-monocyte differentiation. This would be in keeping with the increase in numbers of total colonies, particularly megakaryocyte-containing colonies, observed by *in vitro* culture experiments.

It remains to be determined whether the consequences of genetic perturbations due to trisomy and effects specific to *ERG* trisomy in the context of DS as our data may suggest, could be amenable to therapeutic targeting in the prevention or treatment of DS-TMD or DS-AMKL in addition to currently available strategies.

## Materials and Methods

### Mice

Derivation and genotyping of the *Erg* ^*Mld2*^ mutant allele has been described [21]. Ts(17^16^)65Dn mice (The Jackson Laboratory) were maintained as previously described [20]. All mice were derived from the first-generation progeny of matings between *Erg* ^*+/Mld2*^ and Ts(17^16^)65Dn mice and genotyped as previously described [42]. This study was performed in accordance with the Australian Code for the Care and Use of Animals for Scientific Purposes, published by the Australian National Health and Medical Research Council. Procedures were approved by the Walter and Eliza Hall Institute of Medical Research Animal Ethics Committee (Approval number 2012.003).

### Haematology

Single-cell suspensions from bone marrow were prepared in balanced salt solution (0.15 M NaCl, 4 mM KCl, 2 mM CaCl_2_, 1 mM MgSO_4_, 1 mM KH_2_PO_4_, 0.8 mM K_2_HPO_4_, and 15 mM N-2-hydroxyethylpiperazine-N'-2-ethanesulfonic acid supplemented with 2% [vol/vol] bovine calf serum). Clonal analysis of bone marrow cells (2.5x10^4^) was performed in 1 mL semisolid agar cultures of 0.3% agar in Dulbecco/s modified Eagles medium containing 20% newborn calf serum and stem cell factor; SCF (100 ng/mL), erythropoietin; EPO (2 U/mL), and interleukin-3; IL-3 (10 ng/mL), granulocyte colony stimulating factor; G-CSF (10^3^ U/mL), granulocyte-macrophage colony stimulating factor; GM-CSF (10^3^ U/mL) and/or macrophage colony stimulating factor; M-CSF (10^3^ U/mL). Cultures were incubated at 37°C for 7 days in a fully humidified atmosphere of 10% CO_2_ in air, then fixed, dried onto glass slides, and stained for acetylcholinesterase, Luxol fast blue, and hematoxylin, and the number and type of colonies were determined.

### Flow cytometry

Staining was performed using rat anti-mouse biotinylated or fluorochrome-conjugated antibodies specific for Ter119 (Ly-76), Gr1 (Ly6G and Ly6C), Mac1 (CD11b), B220 (CD45R), CD4, CD8, CD41, CD34, CD16/32, Sca1 (Ly6A/E), cKit (CD117) and CD150 (Biolegend) and IL-7 receptor α (IL7Rα), CD48, CD105, and CD9 (eBioscience). Secondary staining used streptavidin PE-Texas-Red (BD Pharmingen). Cells were analyzed using a LSR Fortessa flow cytometer (Becton Dickinson), or cells were sorted using a FACSAria II (Becton Dickinson) flow cytometer after antibody staining with lineage depletion.

### Statistical analysis

Student's unpaired two-tailed t-tests were used using GraphPad Prism v. 5.0a for Mac Os X (GraphPad Software), unless otherwise specified.

### Microarray expression profiling of murine progenitor cell populations

Bone marrow LSK, CMP, GMP and MEP populations were isolated by FACS from 2–4 mice including males and females from each genotype (*Ts65Dn/Erg* ^*+/+/+*^, *Ts65Dn/Erg* ^*+/+/Mld2*^, *Erg* ^*+/+*^ and *Erg* ^*+/Mld2*^) at ~ 4 months of age. Total RNA was isolated from 100,000–500,000 cells pooled from genotype and cell population matched samples using the RNeasy Micro kit (Qiagen). RNA quality was assessed with the Agilent Bioanalyzer 2100 (Agilent Technologies) by using the Agilent RNA 6000 Nanokit (Agilent Technologies) according to the manufacturer's protocol. Up to 200ng of RNA was labelled with the Total Prep RNA amplification kit (Ambion), and complementary RNA (1.5 μg) was hybridized to 48 arrays using six Illumina Mouse WG-6 V2.0 Expression BeadChips (Illumina, Inc., San Diego, CA) according to Illumina standard protocols. The resultant microarray probe level data were analyzed by using the limma software package Version 3.21.1 [38]. Raw intensities were normalized by using the neqc function, which performs normexp background correction followed by quantile normalization using control probes [43]. Probes were filtered if not detected in any sample (detection *P* value < 0.05). Pairwise comparisons were made by using linear modeling and empirical Bayes moderated t statistics [36]. Empirical array quality weights were estimated and incorporated into the linear models [44]. Allowance was made for possible correlations between RNA samples drawn from the same pool of mice [45]. The false discovery rate (FDR) was controlled by using the Benjamini-Hochberg algorithm. Probes with FDR of less than 5% were considered to be differentially expressed. The microarray data have been deposited to the Array Express database (http://www.ebi.ac.uk/arrayexpress) with accession number E-MTAB-2574.

### RNA-seq expression profiling

Validation of the microarray data was undertaken via RNA-seq. Total RNA was extracted using the RNeasy Plus minikit (Qiagen) from LSK cells sorted independently from three trisomic *Ts65Dn/Erg* ^*+/+/+*^ (two male and one female) and *Erg* ^+/+^ controls (two male and one female) at ~ 4 months of age. Sequencing was performed on an Illumina Hi-Seq 2500, producing at least 15.9 million 100bp paired-end reads per sample. Reads were mapped to the mm10 mouse genome (Genome Reference Consortium GRCm38) using the *Subread* aligner [46]. Read counts were summarised at the gene level by *featureCounts* [47] using NCBI RefSeq gene annotation. Differential expression analysis utilised the *edgeR* [48] and *limma* software packages. Genes were filtered as not expressed if they failed to achieve at least 0.5 counts per million reads in at least 2 of the 6 samples. All Entrez gene IDs without an official symbol were removed from further analysis, as were Y chromosome genes, Xist, and immunoglobulin genes, leaving 14,551 genes for downstream analysis. Library sizes were normalised using the TMM method [49]. The voom function of the limma package was used to convert read counts to log_2_ counts per million with associated precision weights [50]. Differential expression was assessed using empirical Bayes moderated t-statistics [36]. There was a strong correlation between the gene sets identified as differentially expressed by microarray (S2 Table) with RNA-seq data in *Ts65Dn/Erg* ^*+/+/+*^ versus *Erg* ^*+/+*^ LSK cells (ROAST *P* value = 0.00795) and of the differentially expressed genes identified from the microarray comparison, over fifty genes were also identified as differentially expressed using non-pooled independent samples by RNA-Seq with a *P* value of less than 0.05.

### Expression profiling of human Down syndrome cells

Affymetrix microarray CEL files containing gene expression profiles of human DS CD34^+^CD38^-^ HSCs and multipotential progenitors [31] were obtained from the Paterson Institute for Cancer Research at the University of Manchester and analysed with the affy (version 1.34) and limma software packages. Raw intensities were background corrected, normalised and summarized using the Robust Multiarray Average algorithm. Pairwise comparisons were made by using linear modeling and empirical Bayes moderated t statistics.

### Expression signature analyses

Gene sets from the Molecular Signatures Database (Broad Institute, Version 2.5) were mapped from human to mouse orthologs (http://bioinf.wehi.edu.au/software/MSigDB/). Genuine Association analysis [37] used the genas function of the limma package. Rotational gene set tests (ROAST) were performed with the roast function of the limma package, using the “mean” set statistic, array quality weights, Holm modification for multiple testing and 10000 rotations [39]. Moderated t-statistics were used to weight genes in the gene set tests. Barcode plots were made using the barcodeplot function of the limma package.

Heatmaps were plotted using the Heatmap.2 function from the gplots software package, using Pearson correlation for hierarchical clustering for rows and columns. Gene signatures were represented in a heatmap as Z score equivalents. The Z scores were derived from the standard normal distribution and correspond to the continuity-corrected single-tailed *P* values obtained from the ROAST tests, with a positive Z score for an up-regulated gene set and negative Z score for a down-regulated gene set.

## Supporting Information

S1 Table Immunophenotypic definitions of bone marrow stem and progenitor cell populations. (DOCX)

S2 Table Gene expression changes due to full trisomy, attributable specifically to *Erg* trisomy, trisomy of *non-Erg* genes and *Erg* haploinsufficiency in LSK, CMP, GMP and MEP progenitor populations. Differentially expressed probes using a false discovery rate (FDR) of 0.05 as a consequence of full trisomy (pairwise comparison *Ts65Dn/Erg* ^*+/+/+*^ versus *Erg* ^*+/+*^), trisomy of *Erg* (*Ts65Dn/Erg* ^*+/+/+*^ versus *Ts65Dn/Erg* ^*+/+/Mld2*^) and *non-Erg* gene trisomy (*Ts65Dn/Erg* ^*+/+/Mld2*^ versus *Erg* ^*+/+*^) in LSK, CMP, GMP and MEP hematopoietic cells (see [20], Fig 1A). Three biological replicates per cell type per genotype were obtained using pooled RNA derived from 2–4 mice (including male and female) per genotype per replicate, for a total of 48 arrays. See also Figs 2, 3, 5 and Table 2.
(XLSX)

S3 Table Gene signature sets from the curated Broad Institute Molecular Signatures Database version 2.5 (C2 MsigDB) compared against gene expression changes in LSKs as a function of trisomy and trisomy of *Erg* by rotational gene set testing using ROAST. Upregulated genes sets are shown with ROAST *P-*values.
(XLSX)

S4 Table Signature gene sets of murine hematopoietic stem and progenitor populations. An expression signature was defined for each cell population by compiling the significantly up-regulated genes in that cell type compared to the average of all the other populations using a false discovery rate (FDR) of 0.05. See also Fig 4.
(XLSX)

S5 Table Transcriptome analysis of Lin^-^CD34^+^CD38^-^ hematopoietic stem and primitive progenitor cells from human DS bone marrow. See also Fig 5.
(XLSX)

S1 Fig Differential expression of murine orthologs of genes mutated in DS-AMKL: *JAK1, JAK2, JAK3, FLT3, TP53, TRIB1, MPL, EZH2, APC, EXT1 and SMC3*, in *Ts65DnErg* ^+/+/+^ LSK, CMP, GMP and MEP Cells Compared with *Erg* ^+/+^ Cells. Heatmap diagram of log_2_ fold changes of genes expressed in microarray data.
(TIF)
