# Supplementary material for: Early Lineage Priming by Trisomy of Erg Leads to Myeloproliferation in a Down Syndrome Model
Source: PLoS Genet. 2015 May 14;11(5):e1005211. doi: 10.1371/journal.pgen.1005211 (PMC4431731; doi:10.1371/journal.pgen.1005211)
Supplement: S1 Table — (DOCX) [file pgen.1005211.s001.docx]

**Supplementary Table 1. Immunophenotypic definitions of bone marrow stem and progenitor cell populations**

| **Bone Marrow Subset** | **Immunophenotype** |
| --- | --- |
| LSK | Lineage^-^ Sca-1^+^Kit^+^ |
| PreGM, pre-granulocyte macrophage | Lineage^-^IL7Rα^-^cKit^+^Sca1^-^CD150^-^Endoglin^-^FcγRII/III^-^ |
| GMP, granulocyte-macrophage | Lineage^-^IL7Rα^-^cKit^+^Sca1^-^CD150^-^Endoglin^-^FcγRII/III^+^ |
| BEMP, bipotential erythroid megakaryocyte | Lineage^-^IL7Rα^-^cKit^+^Sca1^-^ CD150^+^FcγRII/III^lo^Endoglin^lo^CD9^lo^ |
| CD150^+^CD9^hi^, CD9^hi^ bipotential erythroid megakaryocyte | Lineage^-^IL7Rα^-^cKit^+^Sca1^-^ CD150^+^FcγRII/III^lo^Endoglin^lo^CD9^hi^ |
| CD150^+^ FcγR^+^, FcγRII/III^+^ bipotential erythroid megakaryocyte | Lineage^-^IL7Rα^-^cKit^+^Sca1^-^ CD150^+^FcγRII/III^+^Endoglin^lo^CD9^hi^ |
| PreCFU-E, pre-colony forming unit erythroid | Lineage^-^IL7Rα^-^cKit^+^Sca1^-^ CD150^+^FcγRII/III^-^Endoglin^hi^CD9^lo^ |
| CFU-E, colony forming unit erythroid | Lineage^-^IL7Rα^-^cKit^+^Sca1^-^ CD150^-^FcγRII/III^-^Endoglin^hi^ |
