# Supplementary figures and images for: Early Lineage Priming by Trisomy of Erg Leads to Myeloproliferation in a Down Syndrome Model
Source: PLoS Genet. 2015 May 14;11(5):e1005211. doi: 10.1371/journal.pgen.1005211 (PMC4431731; doi:10.1371/journal.pgen.1005211)

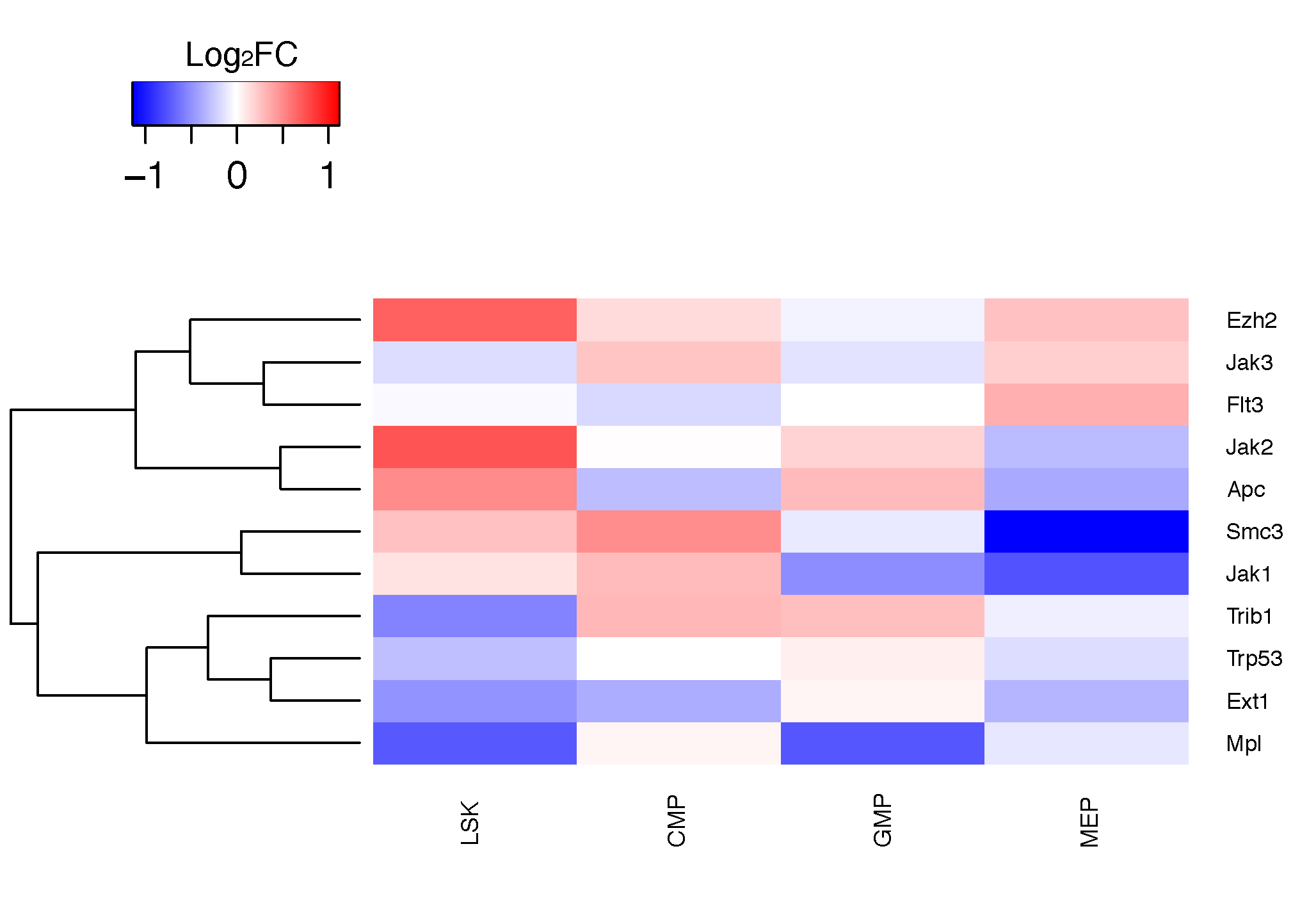

Supplement: S1 Fig — Heatmap diagram of log2 fold changes of genes expressed in microarray data. (TIF) [file pgen.1005211.s006.tif]
